# An Evolutionary Game Theory Model of Spontaneous Brain Functioning

**DOI:** 10.1038/s41598-017-15865-w

**Published:** 2017-11-22

**Authors:** Dario Madeo, Agostino Talarico, Alvaro Pascual-Leone, Chiara Mocenni, Emiliano Santarnecchi

**Affiliations:** 1 University of Siena, Department of Information Engineering and Mathematics, Siena, 53100 Italy; 2 University of Siena, Complex Systems Community, Siena, 53100 Italy; 3 Berenson-Allen Center for Non-Invasive Brain Stimulation, Beth Israel Deaconess Medical Center, Harvard Medical School, Boston, MA USA; 4 University of Siena, Siena Brain Investigation & Neuromodulation Laboratory, Department of Medicine, Surgery and Neuroscience, Neurology and Clinical Neurophysiology Section, Siena, 53100 Italy

## Abstract

Our brain is a complex system of interconnected regions spontaneously organized into distinct networks. The integration of information between and within these networks is a continuous process that can be observed even when the brain is at rest, i.e. not engaged in any particular task. Moreover, such spontaneous dynamics show predictive value over individual cognitive profile and constitute a potential marker in neurological and psychiatric conditions, making its understanding of fundamental importance in modern neuroscience. Here we present a theoretical and mathematical model based on an extension of evolutionary game theory on networks (EGN), able to capture brain's interregional dynamics by balancing emulative and non-emulative attitudes among brain regions. This results in the net behavior of nodes composing resting-state networks identified using functional magnetic resonance imaging (fMRI), determining their moment-to-moment level of activation and inhibition as expressed by positive and negative shifts in BOLD fMRI signal. By spontaneously generating low-frequency oscillatory behaviors, the EGN model is able to mimic functional connectivity dynamics, approximate fMRI time series on the basis of initial subset of available data, as well as simulate the impact of network lesions and provide evidence of compensation mechanisms across networks. Results suggest evolutionary game theory on networks as a new potential framework for the understanding of human brain network dynamics.

## Introduction

The last twenty years of network neuroscience have put forward the idea of the human brain being constantly integrating internal and external stimuli by means of oscillatory dynamics happening at different time and spatial scales, even during so-called “resting-state”^1,2^. Notably, the identification of pivotal characteristics of brain spontaneous activity, e.g. (i) its capacity for simultaneous local and distributed information processing^3,4^, the (ii) organization into separate but integrated networks^5–7^ typically organized in a hierarchical fashion^8,9^, and the (iii) power-law distribution of network nodes importance^3,5,10^, have shown the complexity of brain functioning and its similarity with other biological systems like the immune system^11,12^. Such organization is also responsible for -or the consequence of- individual variability in several traits within cognitive^13–15^ and personality domain(s)^16^; it shows a strong predictive power over evoked activity^17^ and functional alterations when pathological states arise^18–20^. Most importantly, it seems able to capture each individual brain’s uniqueness^21^, making the understanding of its origin a fundamental goal for both theoretical and applied neuroscience^22^.

The principles determining the moment-to-moment local vascular/metabolic supply giving rise to the functional connectivity patterns observed via functional magnetic resonance imaging (fMRI) in the primate and non-primate brain are still under discussion. Several models have attempted at summarizing such organizational principles at different spatial and functional scales. For instance, Izhikevich has proposed models that capture the essence of neuronal behavior in different brain regions, (e.g. cortical, thalamic and hippocampal), looking at the basic rules behind the generations of cortical and subcortical oscillatory pattern^23^. Lewis *et* A*l*. (2010) have built multicellular models focusing on the core of cerebral energy metabolism, including central and mitochondrial metabolic pathways, in order to understand metabolic interactions between various classes of neuron^24^. On a different spatial scale, Tononi and colleagues have suggested the possibility of capturing brain’s complexity by means of a balance in functional integration and segregation^7,25,26^.

Even though all these models are not easily reconcilable in a single framework, they all point towards a definition of the human brain as a complex system far from being organized by means of linear dynamics, in contrast to the vast majority of available models characterizing macroscale spontaneous fMRI dynamics. The analysis of spontaneous and evoked activity recorded using blood oxygenation level dependent (BOLD) signal is mostly based on functional connectivity - inferred by means of linear (e.g Pearson correlation, partial correlation) and nonlinear tools (e.g. mutual infomation)^27,28^ - and effective connectivity (e.g. Granger causality)^29^ metrics to provide a quantification of interregional interplay, while neglecting brain’s non-linear dynamics and stationarity of time series^30^. This might prevent the testing and identification of organizational principles underlying brain’s spontaneous functioning, as those commonly applied to the study of complex network dynamics.

Brain activity can be model according to rules based on the concept of competition and cooperation. Remarkably, such rules have been employed to describe the interaction between populations of neurons^31–33^, and later successfully used in statistical mechanics approaches like spin glass models^34–38^ for the inference of water diffusion in brain tissues during diffusion MRI measurements^39,40^. Statistical mechanics has been also used to describe the activity of nervous nets^41^ by using “replicator equations”, a mathematical tool widely used in the context of evolutionary game theory (EGT)^42–44^. In the context of EGT, here we propose a non-linear model of brain spontaneous activity based on a novel extension of evolutionary game theory allowing the analysis of connectivity dynamics in complex graphs. In mathematics, Game Theory (GT) describes strategic interactions among individuals, where the reward of each player (i.e. any entity representing a node of a complex network, including e.g. social, metabolic and protein networks) depends on both its own and other players’ decisions. However, while GT might explain interactions between players as a process driven by individual benefit, it lacks explanatory power over more complex and counterintuitive dynamics, such as altruism or sexual partner selection^45^. EGT extends this concept by accounting for the dynamics of interactions between individuals in a game-like context driven by evolutionary mechanisms, which take into account – and contribute to explain – dynamics described in classical Darwinian evolution (e.g. competition, natural selection, heredity^42,44^).

In EGT, players are indistinguishable members of a large population, each one characterized by a phenotype which determines their strategy among the *M* available, when playing with any other randomly selected individual of the population. Each player’s payoff earned in the games is evaluated by specific functions, while the system dynamics are described by an ordinary differential equation defined in the *M*-simplex, namely, i.e. the replicator equation^42^. By looking at the evolution of strategies over time, models based on EGT are able to map specific strategies ascribable to *competition* or *coopera* *tion*, allowing to capture spontaneous oscillatory behavior of complex biological and non biological systems. However, EGT only allows to describe the evolution of strategies at the level of the whole population: for example 50% of the population may choose a given strategy while the remaining 50% may choose another, with no information about the behavior of each single node/player. To address these limits, many studies have focused on games played by network populations^46–48^. To address this issue, an extension of EGT on graphs have been recently proposed by our group. In particular, the new model, named Evolutionary Games on Networks (EGN), describes the dynamical interactions of players arranged in a network^49,50^, by taking into account topographical constraints. Crucially, when the balance between competing and cooperating nodes in a given network is considered, the model generates low-frequency oscillatory behaviors that highly resemble those observed in resting-state fMRI data recorded in humans. By applying such model on real fMRI data, the present work demonstrates the possibility to model oscillatory inter-regional brain dynamics by means of EGN, also providing a preliminary evidence of its capacity to generate compensatory dynamics when network lesions are simulated. The following sections will introduce the rationale and specifics of the EGN model for fMRI BOLD data (Evolutionary Games for Brain Networks - EGN-B hereafter), where each brain region, assumed as an assembly of neurons composing anatomically or functionally defined regions, is modeled as a player in an evolutionary game. Under this assumption, we derived a model that incorporates the mechanisms of cooperation and competition among network nodes. The data used for modeling and parametric identification are described as part of the method sections, whereas results and their discussion are presented in a single section to ease the interpretation of findings. Additional details about the model, neuroimaging datasets and fMRI preprocessing are included in Supplementary Information.

## Results and Discussions

### EGN-B model dynamics

Game theory deals with the mathematical modeling of strategic interactions among agents. The result of such interactions is a payoff which an agent receives on the basis of his own selected strategy and and on the strategies of all his opponents. The agent attempts to choose his strategy in order to maximize this payoff.

In the simplest case of two strategies and two players, the payoff is described by means of a payoff matrix:

$${\rm{B}}=[\begin{array}{cc}{b}_{1,1} & {b}_{1,2}\\ {b}_{2,1} & {b}_{2,2}\end{array}],$$

where *b*_*i*,*j*_ is the payoff that player 1 earns when he plays strategy *i* against strategy *j* of player 2. Notably, any payoff matrix can be simplified by considering a diagonal matrix:

$${\rm{B}}=[\begin{array}{cc}\alpha  & 0\\ 0 & \iota \end{array}],$$

where *α* = *b*_1,1_ − *b*_2,1_ and *ι* = *b*_2,2_ − *b*_1,2_^51^, giving rise to an equivalent game. The interpretation of the introduced parameters is straightforward: when *α* > 0 (*ι* > 0), first strategy (second strategy) is preferred by player 1 if player 2 chooses the same strategy. Negative values means instead that player 1 prefers to choose the opposite strategy of player 2. Finally, a null value represents indifference, no strategy is preferred whichever is the choice of the opponent.This simple class of games has been fruitfully used to describe cooperation and competition mechanisms in biology and social sciences, like prisoner’s dilemma and hawk and dove games^44^, and it has inspired us to model the mechanisms (strategies) of activation/inhibition observed during resting-state fMRI acquisitions as games played by different brain areas (i.e. players).

However, activation and inhibition evolves over time. Such dynamics are accounted by evolutionary games theory, which is used to describe the concept of biological selection and evolution, naturally incorporating an optimal decision making mechanism^42,44^. Evolutionary games on graphs^49^ is the extension to populations organized on a graphs, and it represents natural tool to describe brain dynamics since activation and inhibition of brain areas is a dynamical process which takes place over a networked structure.

Let *x*_*v*_(*t*) ∈[0, 1] be the level of activation of area *v* at time *t*: *x*_*v*_(*t*) = 1 stands for fully active, while *x*_*v*_(*t*) = 0 means fully inactive. Conversely, 1 − *x*_*v*_(*t*) reads as the inactivation level of area *v*. For the sake of simplicity, from now on we will drop the dependence of *x*_*v*_(*t*) from time *t*.

Consider a simple case, a brain composed by only two areas *v* and *w*. At each time, *v* checks the level of activity of *w* (*x*_*w*_), evaluates the benefit of activating itself and eventually takes a decision affecting its subsequent level of activity *x*_*v*_. The decision is taken on the basis of activation and inactivation payoffs:

$$\{\begin{array}{ccc}{p}_{v,w}^{A} & = & {\alpha }_{v,w}{x}_{w}\\ {p}_{v,w}^{I} & = & {\iota }_{v,w}(1-{x}_{w})\end{array},$$

where *α*_*v*,*w*_ and *ι*_*v*,*w*_ are activation and inactivation propensities of area *v* with respect to *w* (see the definition of payoff matrix **B**). When the propensities are both positive (*α*_*v*,*w*_ > 0 and *ι*_*v*,*w*_ > 0), area *v* has the attitude to emulate *w*; the activation and inactivation payoffs are proportional to the activation and inactivation levels, *x*_*w*_ and 1 − *x*_*w*_ respectively, of area *w*. Otherwise, area *v* can have a non-emulative attitude described by negative propensities (*α*_*v*,*w*_ < 0 and *ι*_*v*,*w*_ < 0). Propensities with different signs are not considered here since they correspond to unconditional activation (${p}_{v,w}^{A} > {p}_{v,w}^{I}\,\forall {x}_{w}$) or inactivation (${p}_{v,w}^{A} < {p}_{v,w}^{I}\,\forall {x}_{w}$), i.e. the interaction with other areas does not affect the decision process of area *v*.

Given ${p}_{v,w}^{A}$ and ${p}_{v,w}^{I}$, the decision mechanism relies on their difference Δ*p*_*v*,*w*_:

1 $${\rm{\Delta }}{p}_{v,w}={p}_{v,w}^{A}-{p}_{v,w}^{I}=({\alpha }_{v,w}+{\iota }_{v,w}){x}_{w}-{\iota }_{v,w}\mathrm{.}$$

More specifically, *x*_*v*_ should increase when Δ*p*_*v*,*w*_ is positive, i.e. the payoff for activation is bigger than the payoff for inactivation, or should decrease otherwise. As a consequence, an area with emulative attitude should increase its activation level *x*_*v*_ if:

2 $${x}_{w} > {d}_{v}=\frac{{\iota }_{v,w}}{{\alpha }_{v,w}+{\iota }_{v,w}}$$

and should decrease in the other case (i.e. *x*_*w*_ < *d*_*v*_). Conversely, non-emulative attitude should induce an increase of *x*_*v*_ when *x*_*w*_ < *d*_*v*_ and a decrease for *x*_*w*_ > *d*_*v*_. Moreover, the greater is the distance between *x*_*w*_ and *d*_*v*_, the faster should be the change of *x*_*v*_ over time. In the present work we assume that, given an ordered pair of regions (*v*,*w*), the propensities *α*_*v*,*w*_ and *ι*_*v*,*w*_ are both equal to −1 or to +1.

The previous assumptions allow us to write the replicator equation on graphs^49^:

3 $${\dot{x}}_{v}={x}_{v}\mathrm{(1}-{x}_{v})\sum _{w=1}^{N}{a}_{v,w}{\rm{\Delta }}{p}_{v,w},$$

where a_*v*,*w*_ ≥ 0 are the entries of the adjacency matrix A, representing the strength of the influence of an area *w* on the area *v* (a_*v*,*w*_ = 0 if there is no influence). In equation (3), the variation of the activation level *x*_*v*_ over time depends on sum of all payoffs earned through all the interactions of area *v* with neighbors specified by the adjacency matrix A. Equation (3) presents at least two steady states, corresponding to full activation (*x*_*v*_ = 1) and full inactivation (*x*_*v*_ = 0). Far from these points, since *x*_*v*_(1 − *x*_*v*_) is always positive, then the sign of the time derivative ${\dot{x}}_{v}$ corresponds to the sign of ${\sum }_{w\mathrm{=1}}^{N}{a}_{v,w}{\rm{\Delta }}{p}_{v,w}$. Therefore, if the weighted sum of all Δ*p*_*v*,*w*_, which account for the results of all the interactions with neighboring areas, is positive, then area *v* will enforce its level of activation. Otherwise, it will tend to be inactive.

**Figure 1:**
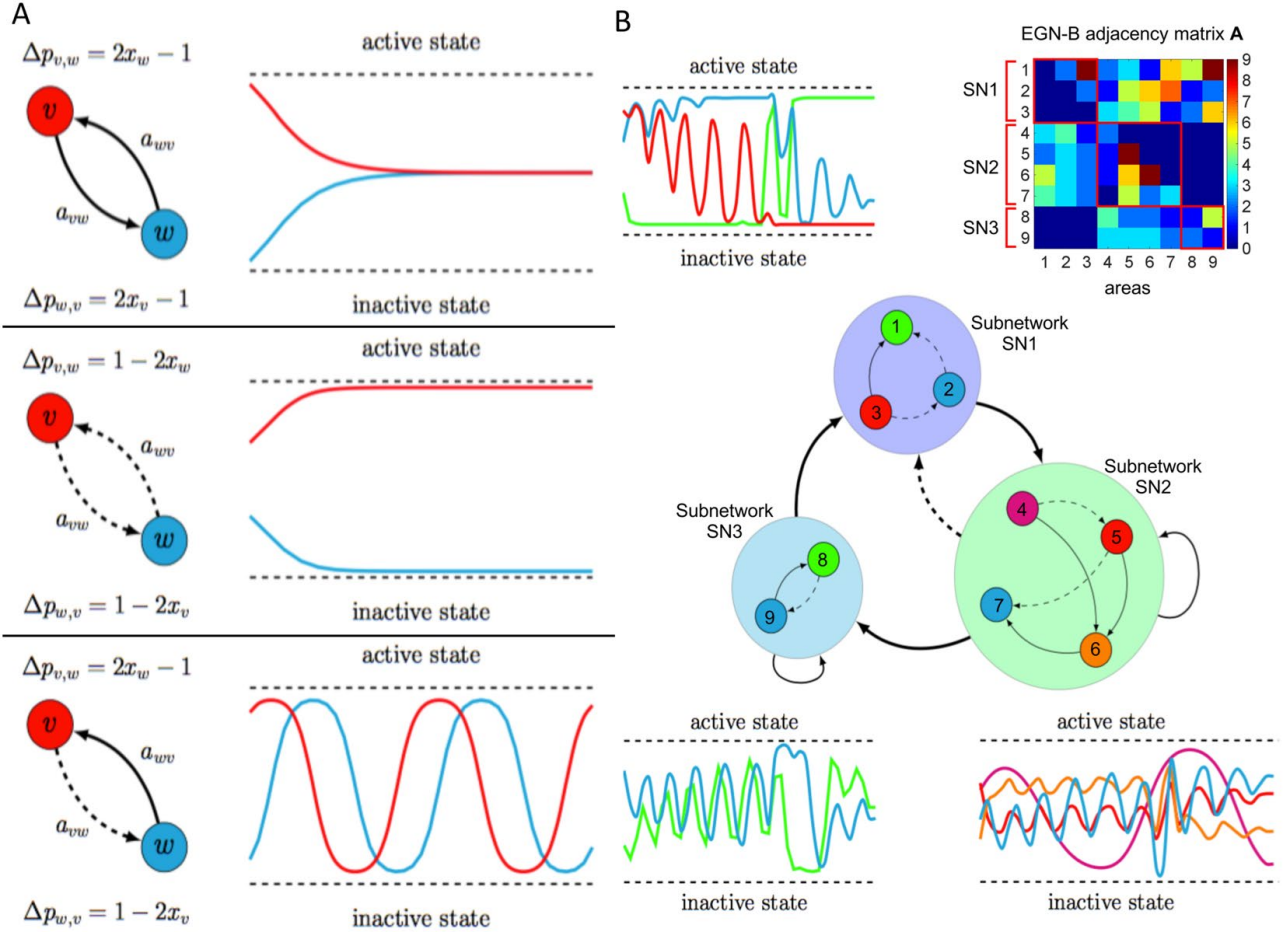
Schematic representation of the mathematical model. (**A**) reports prototypical examples of networks where only two areas are considered, and their corresponding dynamics. The propensities *α*_*v*,*w*_ and *ι*_*v*,*w*_ are set equal to 1 (emulative attitude, solid lines) or to −1 (non-emulative attitude, dashed lines). All the three possible scenarios are shown: both areas converge towards the same level of activation (upper inset), one converges to a fully active state and the other to a fully inactive state (middle inset), and the activation level of both areas are oscillatory (lower inset). (**B**) shows more complex network example including self loops and aggregation of areas. The richness of the dynamics depends on the presence of a higher number of nodes. The EGN-B adjacency matrix A of the network is reported in the upper right corner.

Figure 1 reports some examples of dynamics obtained by solving equation 3. The upper inset of Figure 1A shows the dynamics of two areas both characterized by emulative attitude, driving the two areas to converge to the same activation level. The middle inset depicts two areas with non-emulative attitudes. This mechanism, where the two areas converge to different levels of activation, is referred to as bistability, which is a common phenomenon in brain dynamics^52,53^. Finally, the lower inset shows an oscillating dynamics obtained when the two areas present different attitudes. Figure 1B depicts the dynamics produced using a more complex network of connections and attitudes, as well as clusters of areas. The adjacency matrix A of the EGN-B model is depicted in the upper right corner of Figure 1B.

Notice that the entries a_*v*,*w*_ of the adjacency matrix A, and propensities *α*_*v*,*w*_ and *ι*_*v*,*w*_ are the parameters of the mathematical model (3). Together with the initial conditions, they fully characterize the system dynamics, providing a new layer of knowledge which integrates standard indicators like correlation coefficients, mutual information and Granger causality. More specifically, the EGN-B adjacency matrix A encapsulates information about inter-regional interactions from the point of view of the whole brain, considering this as the best overall configuration/strategy to maximize its evolutionary fitness. Moreover, the signs of parameters *α*_*v*,*w*_ and *ι*_*v*,*w*_ indicates if a given area *v* will emulate or not area *w*. Using the assumptions that for each order pair brain regions (*v*,*w*), we have *α*_*v*,*w*_ = *ι*_*v*,*w*_∈{ − 1, + 1}, then we can introduce the EGN-B signed connectivity matrix A′, whose entries are called connectivity parameters and are defined as follows:

$${a^{\prime} }_{v,w}={a}_{v,w}\cdot {\alpha }_{v,w}\mathrm{.}$$

**Figure 2:**
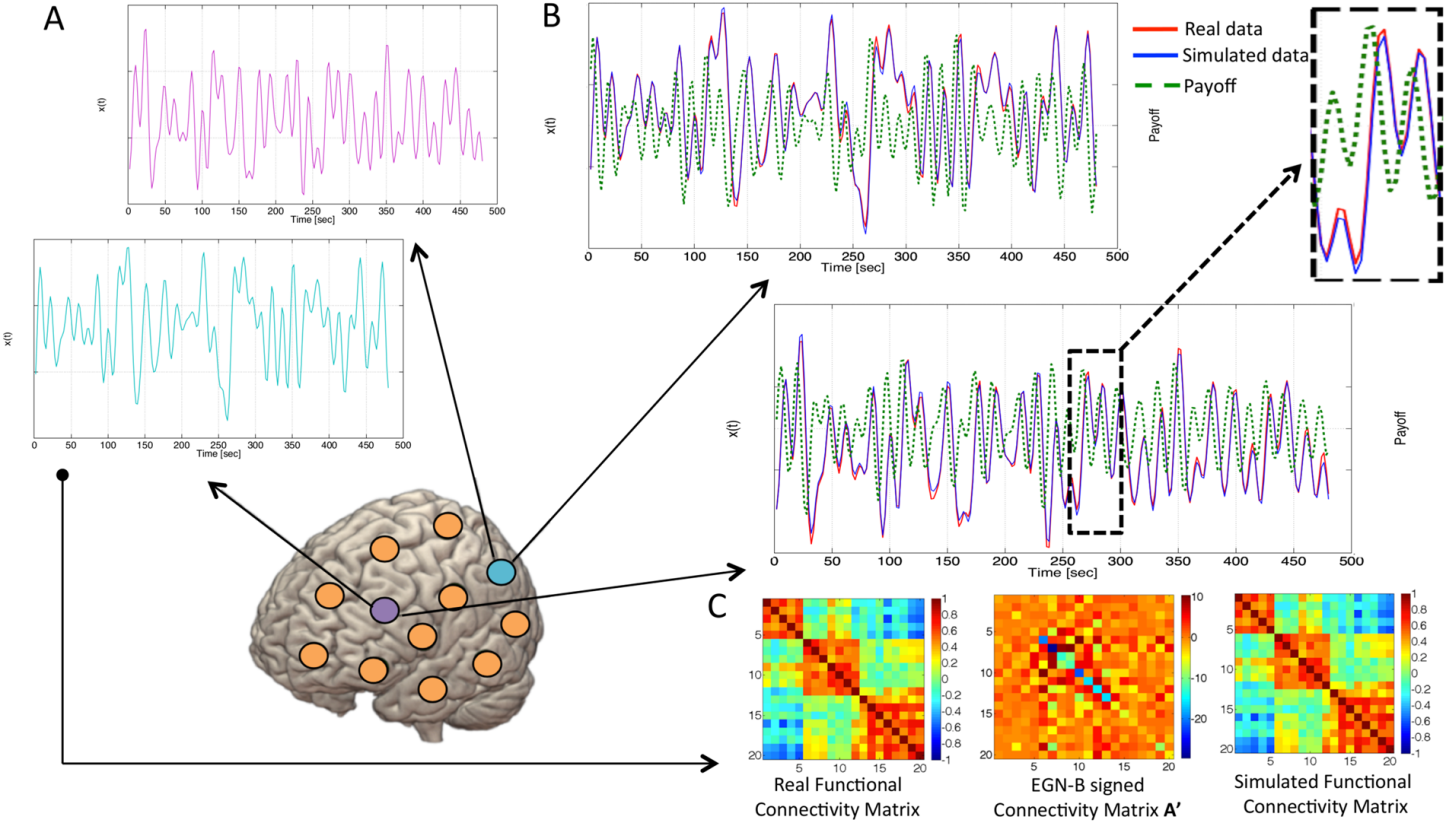
Simulated BOLD signal and brain dynamics. In (**A**) functional MRI data for two illustrative brain regions (cyan and purple dots) are shown, along with the simulated signal obtained using the EGN-B model. (**B**) reports the real fMRI signal (red), the simulated fMRI signal (blue), and the moment-to-moment estimate of dominant strategy for a given node (dashed green line), i.e. the difference between activation and inhibition payoffs (Δ*p*_*v,w*_). The delay between payoff and simulated data represents the predictive power of the model, which is able to determine the most preferable strategy of any network node approximately three seconds ahead of recorded data. In (**C**) are shown a network of 20 nodes composed by regions of interest from the functional atlas published in Dosenbach *et al*. 2010^58^ (left), representing the default mode (node 1–5), visual (node 6–12) and dorsal attention networks (node 13–20), the corresponding EGN-B signed connectivity matrix A′ (center) and the simulated connectivity matrix (right).

It is clear that A = |A′|. An example of the signed matrix A′ is reported in Figure 2C. For further details on matrix A′, refer to Supplementary Information.

The connectivity parameters of the model have been estimated using the resting-state fMRI data described in the subsection Neuroimaging dataset information. Thanks to the linear dependence of EGN-B (3) on these parameters, the identification problem has been solved by means of a linear least square algorithm. The functional to be minimized is defined as:

4 $$F(\theta )=||Y(z)-{\rm{U}}(z)\theta ||,$$

where *z* represents the observed fMRI data, *θ* is the vectorized version of the EGN-B signed functional connectivity matrix A′, and Y(*z*) and U(*z*) are a vector and a matrix, respectively, built up on the basis of a time discretization of the model. The functional in equation (4) is convex and it has only one solution $\hat{\theta }$, namely:

5 $$\hat{\theta }={\rm{\arg }}\mathop{{\rm{\min }}}\limits_{\theta \in {{\mathbb{R}}}^{{N}^{2}}}F(\theta )={({\rm{U}}{(z)}^{{\rm{{\rm T}}}}{\rm{U}}(z))}^{-1}{\rm{U}}{(z)}^{{\rm T}}{\rm{Y}}(z\mathrm{).}$$

More details on model discretization and estimation process can be found in the Supplementary Information.

### Simulating brain activity using EGN-B

Figure 2B shows the fitting between real fMRI data (see Figure 2A) and EGN-B simulated data, produced using the estimated connectivity matrix A′ and the first sample of fMRI recordings as initial condition. Together with data and simulations, time course of the difference between activation and inactivation payoffs Δ*p*_*v,w*_ defined in equation 1 is also reported. It should be noticed that changes of this indicator mimics the variations of the dynamical variable *x*_*v*_ as well as fluctuations in real data happening in the subsequent few seconds. This allows for prediction of BOLD data, and validates the notion of brain’s regions being guided by a continuous balancing between activation and inhibition strategies. Moreover, the estimated matrix A′ is reported in Figure 2C, showing different characteristics of the system when compared to the correlation matrix, hereafter also called FC (functional connectivity matrix). Similarly to the FC matrix, A′ matrix is quasi-symmetric, however depicting a directed graph and describing asymmetric influences among nodes, given that a′_*v*,*w*_ ≠ a′_*w*,*v*_.

Finally, the estimation performance of the EGN-B model has been tested by assuming different network sizes. In particular, we have estimated the adjacency matrix of networks with increasing size, from 2 up to 77 nodes - showing that the average fitting error between real and simulated data decreases with network size (see Figure S1–A in the Supplementary Information). Additionally, the cross-correlation between real and simulated data raises up to the maximum feasible value 1 (see Figure S1–B in the Supplementary Information). Overall, the extraction of a 77 nodes network from the resting-state networks atlas performed in this study guarantees high performance in terms of fitting errors and similarity between real and simulated data. Future studies should investigate such relationship using multiple brain atlases providing even higher spatial resolution, as well as test the performance of EGN-B on both functionally and anatomically-defined brain parcellation schemes.

### EGN-B connectivity and predictive power

**Figure 3:**
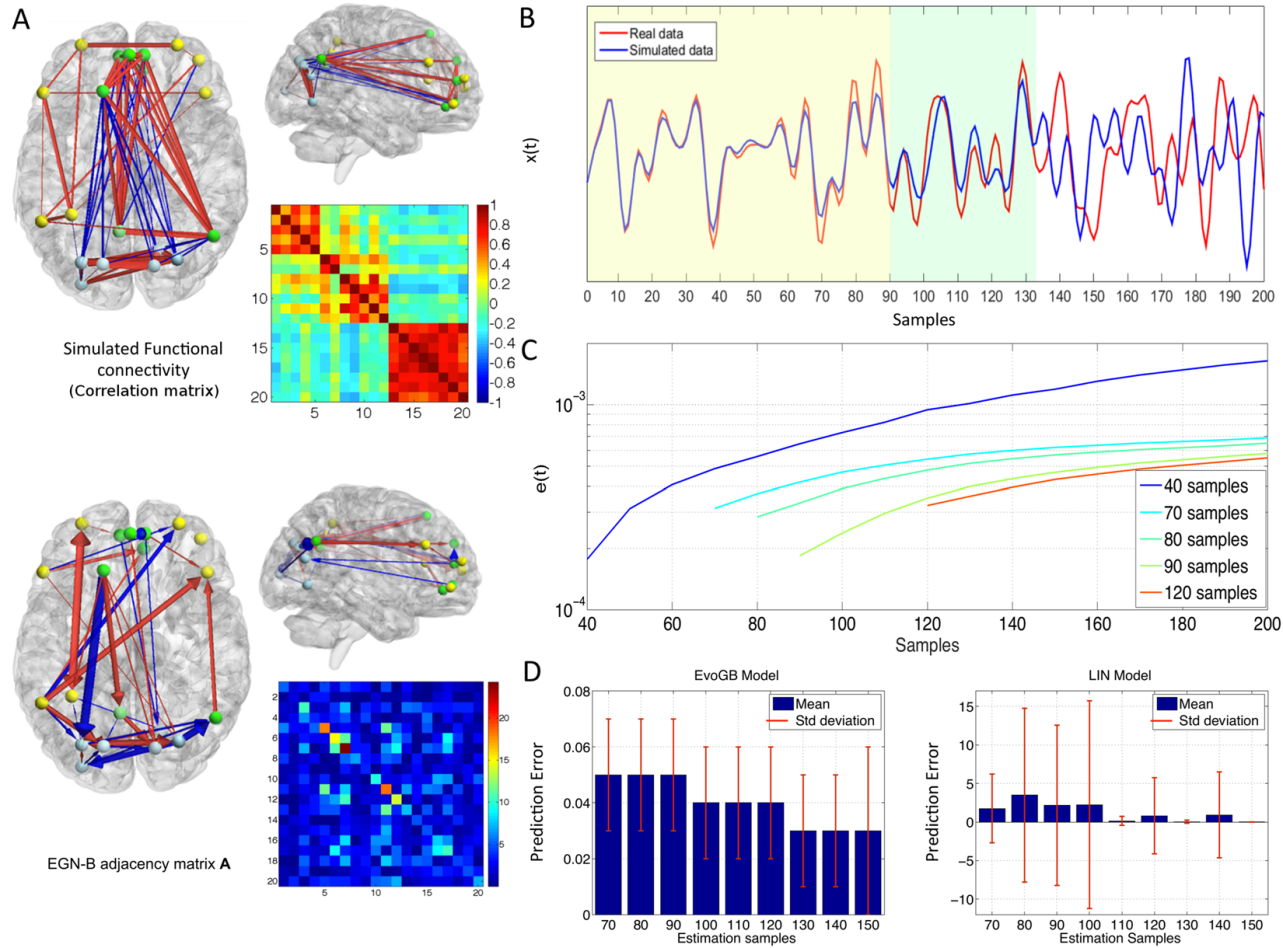
Connectivity, directionality and model errors. (**A**) shows the different networks captured by functional connectivity fMRI analysis based on correlation coefficients (Pearson “r”), and the pattern of activation/inhibition expressed by coefficients of the EGN-B adjacency matrix A. The first captures strong positive within-network correlation, as well as negative ones between nodes of different networks. The EGN-B model unveils a more complex pattern, where positive and negative “directed” modulations are present even within a single network. (**B**) shows the predictive capabilities of the model by comparing real fMRI data of one node (red line) with simulated data (blue line). The latter has been obtained by using an EGN-B connectivity matrix estimated by means of the first 90 real data samples (yellow area). The green area highlights optimal prediction for approximately 45 samples, while prediction accuracy tends to drop thereafter. (**C**) shows the average prediction errors for different size of the estimation dataset across the entire sample. (**D**) depicts the differences between EGN-B and a linear model (reported in the Supplementary Information) in terms of mean and standard deviation of prediction errors in 40 healthy participants.

The difference between functional and EGN-B connectivity is visible in Figure 3A. Figure 3 also reports the performance of the proposed model in terms of temporal prediction of real fMRI data. Specifically, model parameters have been estimated using a subset of available time points (i.e. first 90 fMRI samples) and the entire time series has been then simulated based on previously learned dynamics. Figure 3B shows the comparison between real and simulated data for the whole dataset. The model is able to appropriately predict brain dynamics for approximately 45 samples (≈2 minutes), with a drop in accuracy in subsequent time points. Figure 3C reports the prediction error obtained by estimating parameters using increasing window lengths, with a significant decrease in prediction error when 50% of available time points are considered for model estimation.

Given the theoretical limitations of using linear models to describe brain’s activity, Figure 3D depicts the comparison between performance of EGN-B and a linear model. Following the same procedure as above, the estimation was performed for a subset of data points and remaining time points were then simulated. Hence, error between simulated and real data is evaluated on the predicted time series. Prediction errors show that EGN-B model is more robust than its linear counterpart, with significantly smaller error values (up to 1/100th). In addition, all the simulations errors lie in a small range and have approximately the same standard deviation. On the contrary, prediction errors for the linear model are visibly higher and display an unexpected individual variability among the subjects included in the analysis. The same holds for the standard deviation (see Supplementary Information for a more detailed analysis on the EGN-B and linear model comparison). Interestingly, when the entire set of available time points is used, the model is able to estimate changes in brain dynamics, allowing to reliably predict future network behavior even when changes in network structure are artificially introduced (see Figure S2 in the Supplementary Information). This is particularly relevant when lesions are simulated and the response of single network nodes is modeled.

### Modeling impact of lesions

**Figure 4:**
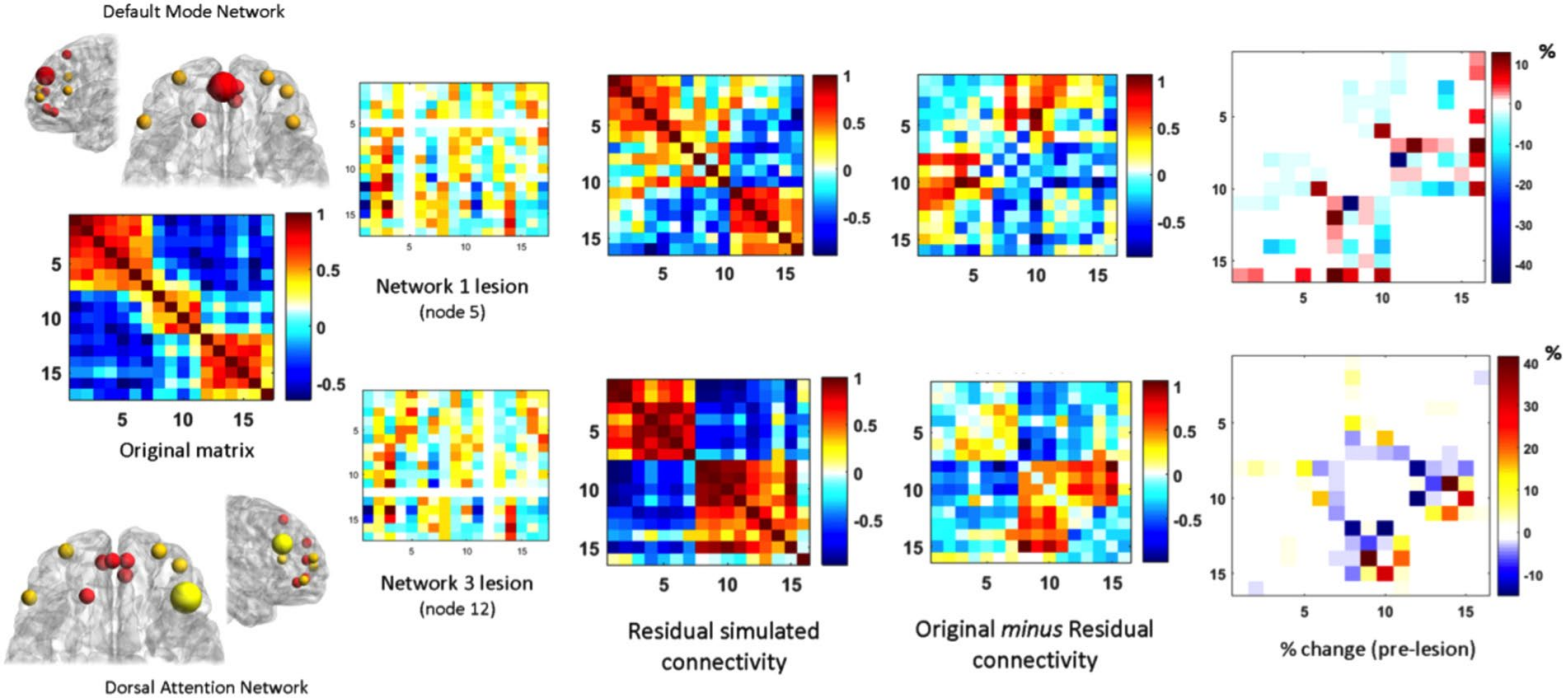
Evolutionary game theory and network response to perturbation. Two distinct lesions to the default mode, dorsal attention and visual networks have been simulated using the EGN-B model. Nodes belonging to the DMN (node 5) and DAN (node 12) have been removed from the adjacency matrix and the resulting connectivity matrix has been estimated using EGN-B. A distinct pattern of rearrangement in connectivity is visible for the two simulations, with changes at both intra and inter-network level. The difference in connectivity strength between original and lesioned (i.e. residual) matrices highlights network-specific response to perturbation, with each lesion inducing differential amount of connectivity increase and decrease depending on the node being targeted (far right).

EGN-B displays the ability to capture and simulate spontaneous BOLD-related brain connectivity patterns, allowing for testing the impact of planned lesions affecting a given resting-state network of interest. Therefore, we additionally tested the response to perturbation of two resting-state networks, namely the default mode (DMN hereafter, composed by 4 nodes,)^1^ and the dorsal attention (DAN hereafter, 7 nodes)^54^ networks. Details about fMRI preprocessing and network extraction are included in the Methods section of the manuscript. The two networks possibly represent the most widely replicated connectivity pattern of the human brain, with their negative correlation^55^ being considered a pivotal characteristic of spontaneous healthy^56^ and pathological brain functioning^57^. The two networks support different cognitive dynamics, with the DAN being involved in attention-related processes (e.g. directing attention towards salient stimuli, filtering) and the DMN being related to mind-wandering and memory. Even though the understanding of their response to perturbation might have important clinical applications, the present analysis only focused on modeling their BOLD-related dynamics. Node - and corresponding connections - removal modeled via EGN-B induced differential changes in brain connectivity. Specifically, lesion to nodes of the DMN and DAN seems to induce different alterations of within and between-network dynamics. Lesion of the medial prefrontal cortex in the DMN does not affect within-network dynamics, while a change in the magnitude of the negative correlation with the Visual network (VN hereafter) is visible. Differently, lesion to the right middle prefrontal cortex node of the DAN markedly switches the connectivity between DAN and VN, also inducing an increase in VN intrinsic connectivity. Interestingly, lesions also differed in terms of magnitude and sign of the observed effects: lesion to the DMN predominantly lead to a decrease in connectivity strength (−43%), with increases in connectivity reaching a maximum of 12% of the original correlation strength (Figure 4). Conversely, lesion to the DAN induced a mirrored effect, with increases in connectivity up to 41% and decreases equal to −13%. Overall, the EGN-B seems able to capture residual brain dynamics beyond spontaneous functioning. However, given the simplified nature of the example discussed here, findings might be interpreted carefully. For instance, the apparent symmetry in the response to lesion to the DMN and DAN (−43% and + 12%; + 41% and −13%) might be due to the limited dynamics being sampled: for the sake of simplicity, each network has been reduced to a lower number of nodes respect to what is measurable with resting-state fMRI data; this could dramatically decrease the complexity of response to perturbation, maximizing the loading on the dominant networks in the matrix (i.e. DMN, DAN). However, it must be noticed that the topography of the two responses show high dissimilarity, with a different involvement of the third (not-lesioned) network.

While lesioning procedure was not the main focus of the present investigation, future studies should test EGN-B on more complex matrices, capturing the dynamics of the entire set of well-known resting-state networks^58–60^. Recent investigations have tested the impact of lesion on functional connectivity by using agent-based models^61^, graph theory^62,63^ and network control theory^64^. Importantly, it must be noticed that a lesioning procedure like the present one only captures a “static” response to external perturbation, without providing any information about dynamic rearrangement in spontaneous BOLD fluctuations evolving over time. EGN-B might expand this scenario by providing non-linear estimates of such changes, which might be of relevance when investigating the effects of, for instance, non-invasive brain stimulation approaches done for both cognitive enhancement purposes^65,66^ or to probe brain plasticity levels in clinical^67^ and cognitive^68^ contexts. EGN-B capacity to predict and model brain response to lesions should also be tested against other widely used analytical tools, like those applied to financial credit networks (to model response to financial default of banks^69^) or the immune network (idiotypic network formation during onthogenesis through positive/negative selection^70^).

### Evolutionary dynamics in the brain

The present data demonstrate how principles and mechansims of evolutionary game theory might be able to capture the complexity of spontaneous, rhythmic brain activity. Even though this constitutes the first evidence of such application, the replicator equation has been used to describe several other biological phenomena, such as evolution driven by replication-selection^43^, mutation^44,71^, tumor progression^72^, bacterial network formation^73^, evolution of opinions^74^, cooperation^44^, political orientations^75^ and consensus control^76^, supporting its capacity to represent complex biological and non-biological dynamics. Results also highlight the importance of looking at self-emerging evolutionary trajectory rather than static game theoretical metrics, suggesting intriguing parallelism with principles and concepts pertaining to the field of evolutionary biology and physiology^77,78^. In all the above mentioned applications, fitness represents a performance measure of a specific given strategy. In the context of fMRI connectivity analysis, we assume each brain region acting as a player in an evolutionary game, activating or inhibiting itself, "following" rules based on cooperation and competition among regions. Here we argue that such balance in competition and cooperation - as captured by EGN-B - might be the optimal strategy for pursuing multiple non mutually exclusive goals related to moment-to-moment metabolic demands: (i) guarantee the needed supply of energy to each brain region in the context of a fixed-resources environment, and (ii) allow synchronization of brain regions belonging to specific networks, while also (iii) avoiding massive co-activation at whole brain level which might represent an excessive metabolic cost. However, the argument of neuroenergetics goes against the notion of brain resources being always at neurons’ disposal (e.g. glycogen stored in astrocytes), with no real limitation in energy supply whatsoever. Yet representing only 2% of the total body mass, the brain consumes about 20% of the oxygen and 25% of the glucose available to the human body^1^, making the need for a perfectly balanced system less likely to be the case. An alternative view might take into account the optimization of information processing, which implies the ability to allocate resources across multiple functional systems in real time. Nevertheless, this implies that brain spontaneous activity also represents a continuous prediction of upcoming sensorial stimuli, a view which does not reflect the canonical definition of BOLD activity (i.e. mostly reflecting local instantaneous activity)^79–81^ and does not include any element of “prediction” with respect to other brain regions’ behavior, thus not fitting with an evolutionary game theory design. However, recent evidence based on optical imaging in monkeys is challenging the nature of BOLD signal, suggesting that activity measured via fMRI might carry at least two types of information, related to local neuronal activity and, notably, anticipatory - or preparatory - task-related activity^82^. The adaptive, predictive component of BOLD signal might allow to infer which brain regions will require additional metabolic resources in the near future, therefore implying an ongoing balancing between regions activation and inhibition which may be mislabeled as a local phenomenon. This view has been also linked to the neuro-hemodynamics hypothesis (see the “hemo-neural” hypothesis in^83^), making them two non-mutually exclusive interpretations.

## Methods

### Neuroimaging dataset information

Data were part of a multimodal data collection which includes a broad phenotypical characterization of 160 healthy subjects (age 19 to 45 years), as well as structural (anatomical and DTI) and functional (resting-state fMRI) neuroimaging data. A selection of subjects was performed to ensure (i) an age range of 19–25 years old (healthy, young adult), (ii) a balance between gender distribution, and (iii) that all subjects were right-handed. The selection resulted in a final sample of 84 right-handed subjects (41 males), with mean age of 29,54 years (standard deviation (SD) = 11). The institutional review boards of the University of Siena (Siena, Italy) and Beth Israel Medical Deaconess Center (Boston, MA, USA) approved the receipt and dissemination of the data. The present study has been approved by the ethical committee of “Policlinico Le Scotte” (Siena, Italy), and it has been performed in accordance with the relevant guidelines and regulations. Informed consents have been obtained from all the human participants of the study. Details about specific MRI sequences and data analysis are reported below.

### Resting State fMRI

Our brain is a complex system of interconnected regions spontaneously organized into distinct networks^30,55,84^. Such intrinsic organization of spontaneous brain activity is captured within the framework of brain connectivity analysis^85^. Differently from canonical task-fMRI paradigm where brain signal of interest is derived by contrasting subject’s activity during an active (e.g. a cognitive task) and a passive state, this approach relies on endogenous brain oscillations recorded during spontaneous brain activity, giving rise to temporally and spatially independent resting state networks^22^. Such methodology has been proven to hold enough information to allow the identification of pathological conditions (e.g. multiple sclerosis^86^, schizophrenia^87^ and Alzheimer^88^) as well as to identify correlates of several cognitive^13,14,89^ and psychological traits^16^ in healthy humans. Here we considered data collected during resting-state, while participants were asked to lay in the MRI scanner with their eyes opened, fixate a cross-air and mind-wander without focusing on any particular topic.

### fMRI Data Preprocessing

Information about the neuroimaging data are included as part of the Supplementary Information of the paper. Functional image preprocessing was carried out using SPM8 software (Statistical Parametric Mapping; http://www.fil.ion.ucl.ac.uk/spm/) and MATLAB 7.5 (MathWorks, MA, USA). The first five volumes of functional images were discarded for each subject to allow for steady-state magnetization. EPI images were slice-time corrected using the interleaved descending acquisition criteria, and realigned and re-sliced to correct for head motion using a mean functional volume derived from the overall fMRI scans. Subject whose head motion exceeded 1.0 mm or rotation exceeded 1.0° during scanning were excluded. In order to obtain the better estimation of brain tissues maps, we implemented an optimized segmentation and normalization process using DARTEL (Diffeomorphic Anatomical Registration using Exponentiated Lie Algebra)^90^ module for SPM8. Briefly, this approach is based on the creation of a customized anatomical template built directly from participants T1-weighted images instead of the canonical one provided with SPM (MNI template, ICBM 152, Montreal Neurological Institute). This allows a finer normalization into standard space and consequently avoids under - or over - estimation of brain regions volume possibly induced by the adoption of an external template. Hidden Markov Random Field model was applied in all segmentation processes in order to remove isolated voxels^91^. Customized tissue prior images and T1-weighted template were smoothed using an 8 mm full-width at half-maximum (FWHM) isotropic Gaussian kernel. Functional images were consequently non-linearly normalized to standard space and a voxel resampling to (isotropic) 3 × 3 × 3 mm were applied. Linear trends were removed to reduce the influence of the rising temperature of the MRI scanner and all functional volumes were band pass filtered at (0.01*Hz* < *f* < 0.08*Hz*) to reduce low-frequency drift. Finally, a CompCor algorithm has been applied in order to control physiological high-frequency respiratory and cardiac noise^92^.

### Lesioning procedure

Even though measuring brain activity is fundamental to understand both human behavior and pathological conditions, a very informative approach resides in the measurement of brain’s ability to cope with external (e.g. concussion, anesthesia, noninvasive brain stimulation) and internal (e.g. brain tumor, stroke) perturbations^68,93^. Being able to predict brain’s response to these events could provide important information for tailoring rehabilitation programs, as well as to move towards individualized interventions by leveraging individual’s resilience profile. Given EGN-B model’s ability to capture and simulate spontaneous BOLD-related brain dynamics, we additionally tested the response to perturbation of two resting-state functional connectivity networks. The networks, representing the default mode (DMN hereafter, 4 nodes,)^1^ and dorsal attention (DAN hereafter, 7 nodes)^54^ networks, were obtained by applying the preprocessing routine described in the Supplementary Information of the manuscript. A third network, the visual network (VN), was included to evaluate cascade effects outside the networks being targeted (DMN, DAN). Single node removal was modeled and network response was simulated using EGN-B. The lesioning procedure was done by (i) modeling the original intact network using EGN-B, (ii) calculating the adjacency matrix, (iii) removing a given target node, and (iv) modeling the response of the network by simulating spontaneous oscillatory activity of each node in the network over time. Simulated BOLD time series have been created with a length equal to the original BOLD time series. Two different nodes were removed, respectively those showing highest within-network connectivity values in the DMN and DAN. Simulations were run on individual MRI data and average at the group level (*N* = 82). Results depict the average rearrangement observed across individuals when the abovementioned lesions are modeled. Changes in network connectivity were reported as the average difference in the functional connectivity (FC) between the original and lesioned (i.e. simulated using EGN-B) connectivity matrices, as well as percentage of changes respect to original (pre-lesion) FC coefficients.

### Data availability

The datasets generated during and/or analysed during the current study are available from the corresponding author on reasonable request.

## Conclusion

The complexity of spontaneous brain functioning might follow the same evolutionary game theory principles that regulate fitness in many other complex biological systems. This implies a self-emergent structure based on a continuous balancing between each brain’s region tendency to imitate or compete against other regions, which give rise to inter-regional dynamics different from what usually observed with functional connectivity analysis. Non-linear estimates of causality between network nodes and BOLD signal prediction might provide new insight in brain functioning and help understanding the origin and substrates of brain’s organizational efficiency.

## Electronic supplementary material

Supplementary Information
