# Supplementary material for: An Evolutionary Game Theory Model of Spontaneous Brain Functioning
Source: Sci Rep. 2017 Nov 22;7:15978. doi: 10.1038/s41598-017-15865-w (PMC5700053; doi:10.1038/s41598-017-15865-w)
Supplement: Supplementary file 1 — Supplementary Information [file 41598_2017_15865_MOESM1_ESM.pdf]

An Evolutionary Game Theory Model of Spontaneous Brain Functioning

Dario Madeo[1,2,*], Agostino Talarico[1], Alvaro Pascual-Leone[3], Chiara Mocenni[1,2], Emiliano Santarnecchi[3,4]

[1] University of Siena, Department of Information Engineering and Mathematics, Siena, 53100, Italy

[2] University of Siena, Complex Systems Community, Siena, 53100, Italy

[3] Berenson-Allen Center for Non-Invasive Brain Stimulation, Beth Israel Deaconess Medical Center, Harvard Medical School, Boston MA, USA

[4] University of Siena, Siena Brain Investigation & Neuromodulation Laboratory, Department of Medicine, Surgery and Neuroscience, Neurology and Clinical Neurophysiology Section, Siena, 53100, Italy

[*] madeo@dii.unisi.it

Supplementary Information (SI)

Data acquisition

MRI data was acquired on a 1.5 T (Philips Intera) whole-body scanner. Resting-state fMRI data included 178 volumes with 33 axial slices covering the whole brain, acquired via a T2 BOLD-sensitive multi-slice echo planar imaging (EPI) sequence ($TR/TE = 2.5s/32\ ms$; field of view= 22 cm; image matrix = 64×64; voxel size = $3.44.44 \times 3.8\ mm^3$; flip angle = $75°$). Structural imaging was performed using a whole brain T1-weighted Fast Field Echo 1 mm^3 sequence ($TR/TE = 30/4.6\ ms$, field of view = 250 mm, matrix 256×256, flip angle = $30°$, slice number = 150). T2-weighted Fluid Attenuated Inverse Recovery Images (FLAIR) were also acquired to assess participants white matter integrity. Participants were provided with earplugs. Particular care was taken to minimize head motion via vacuum cushions and custom made padding. Both structural and fMRI data have been carefully checked for data quality.

fMRI data preprocessing

Functional image preprocessing and statistical analyses were carried out using SPM8 software (Statistical Parametric Mapping; http://www.fil.ion.ucl.ac.uk/spm/) and MATLAB 7.5 (MathWorks, MA, USA). The first five volumes of functional images were discarded for each subject to allow for steady-state magnetization. EPI images were slice-time corrected using the interleaved descending acquisition criteria, and realigned and re-sliced to correct for head motion using a mean functional volume derived from the overall fMRI scans. Subject whose head motion exceeded $1.0mm$ or rotation exceeded $1.0°$ during scanning were excluded. In order to obtain the better estimation of brain tissues maps, we implemented an optimized segmentation and normalization process using DARTEL (Diffeomorphic Anatomical Registration using Exponentiated Lie Algebra) [1] module for SPM8. Briefly, this approach is based on the creation of a customized anatomical template built directly from participants T1-weighted images instead of

the canonical one provided with SPM (MNI template, ICBM 152, Montreal Neurological Institute). This allows a finer normalization into standard space and consequently avoids under - or overestimation of brain regions volume possibly induced by the adoption of an external template. Hidden Markov Random Field model was applied in all segmentation processes in order to remove isolated voxels [2]. Customized tissue prior images and T1-weighted template were smoothed using an 8mm full-width at half-maximum (FWHM) isotropic Gaussian kernel. Functional images were consequently non-linearly normalized to standard space and a voxel resampling to (isotropic) $3 \times 3 \times 3$ mm were applied. Linear trends were removed to reduce the influence of the rising temperature of the MRI scanner and all functional volumes were band pass filtered at $(0.01Hz < f < 0.08Hz)$ to reduce low-frequency drift. Finally, a CompCor algorithm has been applied in order to control physiological high-frequency respiratory and cardiac noise [3].

EGN-B Model

The model proposed in this paper assumes that each agent is an aggregated (brain area) of interacting elementary components (neurons). Each area is assumed to be able to make decisions, and it is characterized by its activity level or state. The state, belonging to the set $[0, 1]$, quantifies the activity level of the area at a particular time. Dynamically, each area compares its activity level with others and it changes its state accordingly, in order to maximize a certain payoff function based on activation and inactivation strategy mechanisms defined below.
The above mechanism is naturally embedded into the replicator equation on graphs [4], describing the EGN-B model as follows:

$$\dot{x_v} = x_v(1 - x_v) \sum_{w=1}^{N} a_{v,w} \Delta p_{v,w} \tag{1}$$

where:

$$\Delta p_{v,w} = p^A_{v,w} - p^I_{v,w},$$
$$p^A_{v,w} = \alpha_{v,w} x_w,$$
$$p^I_{v,w} = \iota_{v,w}(1 - x_w).$$

$p^A_{v,w}$ and $p^I_{v,w}$ represent the payoff of activation and inactivation, respectively. Moreover, $\alpha_{v,w}$ and $\iota_{v,w}$ are the activation and inactivation propensities of payer v with respect to player w. Finally, $x_v \in [0, 1]$ is the level of activation of the brain area v.

Estimation method

In order to characterize the system (1), we must estimate $3N^2$ parameters, namely $a_{v,w}$, $\alpha_{v,w}$ and $\iota_{v,w}$, using the available observed data (the fMRI recordings correspond to the behavior of particular brain areas). The estimation procedure is constrained: in fact, the entries of adjacency matrix $a_{v,w}$ must be non-negative, while the propensities $\alpha_{v,w}$ and $\iota_{v,w}$ must have the same sign for each ordered couple (v, w). In this work we relax these constraints in order to tackle with an unconstrained optimization problem. To this aim, we set $\alpha_{v,w} = \iota_{v,w}$; in this way, the threshold d_v is set to 0.5. Moreover, since the parameters $a_{v,w}$ quantify the strength of connections, then we assume that $\alpha_{v,w}$ and $\iota_{v,w}$ belong to the set $\{-1, +1\}$. Moreover, parameters $\alpha_{v,w}$ and $\iota_{v,w}$ in

(1) are always multiplied by the non-negative parameters $a_{v,w}$, thus affecting the identifiability of the model. For these reasons, we can instead estimate the following N^2 parameters:

$$a'_{v,w} = a_{v,w}\alpha_{v,w} = a_{v,w}\iota_{v,w}.$$

Since $\alpha_{v,w} = \iota_{v,w} \in \{-1, 1\}$, the parameters reads as:

$$a'_{v,w} = a_{v,w}\mathrm{sign}(\alpha_{v,w}) = a_{v,w}\mathrm{sign}(\iota_{v,w}).$$

The result of the estimation method is represented by the matrix $\mathbf{A}' = \{a'_{v,w}\} \in \mathbb{R}^{N\times N}$ which simultaneously contains two important informations:

- since $\mathbf{A} = |\mathbf{A}'|$, it encapsulates the network structure;
- since $\mathrm{sign}(a'_{v,w}) = \mathrm{sign}(\alpha_{v,w}) = \mathrm{sign}(\iota_{v,w})$, it describes all the possible game interactions, such as emulative ($\alpha_{v,w} = \iota_{v,w} > 0$) and non-emulative game ($\alpha_{v,w} = \iota_{v,w} < 0$).

Notice that, if $a'_{v,w} = 0$, then w does not influence v. On the contrary, if $a'_{v,w} \neq 0$, then $|a'_{v,w}|$ denotes the strength of their interaction, while $\mathrm{sign}(a'_{v,w})$ denotes if both activation and inhibition propensities are positive or negative.

Starting from these premises, we can rewrite the EGN-B in (1) as follows:

$$\dot{x}_v = x_v(1 - x_v)f_v(x, \mathbf{A}') \tag{2}$$

where

$$\begin{aligned} f_v(x, \mathbf{A}') &= \sum_{w=1}^{N} a_{v,w}[\alpha_{v,w}x_w - \iota_{v,w}(1 - x_w)] = \\ &= \sum_{w=1}^{N} [a'_{v,w}x_w - a'_{v,w}(1 - x_w)] = \\ &= \sum_{w=1}^{N} a'_{v,w}(2x_w - 1). \end{aligned}$$

Last equation shows that the system (2) linearly depends on the parameters of the signed EGN-B connectivity matrix $\mathbf{A}'$, and it can be rewritten in an alternative form:

$$\dot{x}_v = u_v(x)^\top \theta_v, \tag{3}$$

where $u_v(x) = x_v(1 - x_v)[2x_1 - 1, 2x_2 - 1, \ldots, 2x_N - 1]^\top$, $\theta_v = [a'_{v,1}, a'_{v,2}, \ldots, a'_{v,N}]^\top$ and $x = [x_1, x_2, \ldots, x_N]^\top$.

Moreover, the model (3) can be discretized using the Euler method with time step τ_S:

$$y_v(t_{k+1}) = u_v(x(t_k))^\top \theta_v, \tag{4}$$

where $x_v(t_k)$ is the state variable at time $t_k = k\tau_S$, $y_v(t_{k+1}) = \tau_S^{-1}(x_v(t_{k+1}) - x_v(t_k))$ is the approximation of the time derivative $\dot{x}_v$, and τ_S is the sampling time.

According to (4), the parameters of the model (i.e. the entries of the signed EGN-B connectivity matrix $\mathbf{A}'$) can be estimated using a least square optimization approach. In particular, we

indicate with $z_v(t_k)$ the observed fMRI data collected with sampling time τ_S for T time instants. By substituting $x_v(t_k)$ with $z_v(t_k)$ for each $k \in \{0, 1, \ldots, T-1\}$, we can introduce the vector:

$$Y(z) = \begin{bmatrix} y_1(t_1) \\ y_2(t_1) \\ \vdots \\ y_N(t_1) \\ y_1(t_2) \\ y_2(t_2) \\ \vdots \\ y_N(t_2) \\ \vdots \\ y_1(t_T) \\ y_2(t_T) \\ \vdots \\ y_N(t_T) \end{bmatrix}$$

and the matrix

$$\mathbf{U}(z) = \begin{bmatrix} u_1(z(t_0))^\top & 0\ldots0 & \ldots & 0\ldots0 \\ 0\ldots0 & u_2(z(t_0))^\top & \ldots & 0\ldots0 \\ \vdots & \vdots & \ddots & \vdots \\ 0\ldots0 & 0\ldots0 & \ldots & u_N(z(t_0))^\top \\ u_1(z(t_1))^\top & 0\ldots0 & \ldots & 0\ldots0 \\ 0\ldots0 & u_2(z(t_1))^\top & \ldots & 0\ldots0 \\ \vdots & \vdots & \ddots & \vdots \\ 0\ldots0 & 0\ldots0 & \ldots & u_N(z(t_1))^\top \\ \vdots & \vdots & \vdots & \vdots \\ u_1(z(t_{T-1}))^\top & 0\ldots0 & \ldots & 0\ldots0 \\ 0\ldots0 & u_2(z(t_{T-1}))^\top & \ldots & 0\ldots0 \\ \vdots & \vdots & \ddots & \vdots \\ 0\ldots0 & 0\ldots0 & \ldots & u_N(z(t_{T-1}))^\top \end{bmatrix},$$

thus obtaining the following matricial form:

$$Y(z) = \mathbf{U}(z)\theta, \tag{5}$$

where $\theta = [\theta_1^\top, \theta_2^\top, \ldots, \theta_N^\top]^\top$. Finally, we can estimate the parameters θ by solving the following problem:

$$\hat{\theta} = \arg\min_{\theta \in \mathbb{R}^{N^2}} \|Y(z) - \mathbf{U}(z)\theta\|, \tag{6}$$

Functional in equation (6) is convex and, under certain hypotheses (see *Technical issue and remarks* paragraph at the end of this section), it has only one solution:

$$\hat{\theta} = (\mathbf{U}(z)^\top \mathbf{U}(z))^{-1} \mathbf{U}(z)^\top Y(z), \tag{7}$$

where $\hat{\theta}$ are the estimated parameters based on the observation z.

Notice that the state variables of the dynamical model involved in this work are bounded in the set $[0, 1]$. For this reason, data before interpolation have been normalized in the set $[0.3, 0.7]$.

Technical issue and remarks Notice that $Y(z) \in \mathbb{R}^{NT}$ and $\mathbf{U}(z) \in \mathbb{R}^{NT \times N^2}$. Then $(\mathbf{U}(z)^\top \mathbf{U}(z))^{-1}$ is well-defined if $\text{rank}(\mathbf{U}(z)) = N^2$. For this reason, it is a good practice to set $T > N$, thus allowing $\mathbf{U}(z)$ to have more rows than columns; this increases the probability to deal with a well-posed problem. Unfortunately, the number of available measurements is often small; fMRI data scan makes sense if the subject remains in the identical position for the whole duration of the experiment, that can persist in the interval $[5, 10]$ minutes at a sampling time in the range $[2, 3]$ seconds. Anyway, the bandwidth of fMRI signals is $B = 0.1$ Hz and the measurements in this work are sampled with a frequency $\frac{1}{\tau_S} = \frac{1}{[2,2.5]} = f_S \in [0.4, 0.5]$ Hz. Thanks to the Nyquist-Shannon theorem, since the $f_S \geq 2B$ all the frequency information of signals are kept after the sampling process. Hence we can interpolate the available data in order to have a finer time discretization, thus increasing the value of T up to the desired target. In this work, we have interpolated the fMRI signals by using splines, adding 100 samples between each couple of time consecutive real data.

We remark that the dimensionality of the problem can become a problem as long as the size of the considered network increases [5]. In order to avoid identification problems when dealing with huge networks, one can imagine a multiple steps identification where the weak connections are removed in order to reduce the size of the problem using a priori information like the knowledge of physical and anatomical connectivity.

However, a sufficiently large number of nodes is required to guarantee satisfactory model performances. To show this, we considered several different size models, increasing from 2 to 77 nodes. This investigation has been performed for all the subjects involved in the study. Thereafter, for each estimated model, we computed the average fitting error as a function of the size of the network, as depicted in Figure S1-A. Notably, as long as network size increases, the fitting error decreases, thus improving the model performances. Moreover, the cross correlation between real and simulated data for each network size has been evaluated. In Figure S1-B, we report the minimum (cyan) and the maximum (magenta) values of the cross-correlation curves. We observe that the min/max cross-correlation range increases as the number of the network nodes increases. Furthermore, when the network size is sufficiently large (> 65 nodes), both minimum and maximum cross-correlation values are positive, thus combining the high fitting model performances with a strong similarity between simulated and real data. Finally, for more than 70 nodes, the cross-correlation raises to 1, making reasonable the extraction of the 77 nodes network from the atlas performed in this study.

Comparison with linear systems

In order to evaluate the performances of the proposed EGN-B model, we performed a comparison with standard linear systems. Firstly, for each subject in the available dataset we estimated the EGN-B adjacency matrix using a number of estimation samples N_E lower than the number of samples of the full recording of a single subject (240 samples). Thereafter, we simulated the model using the estimated adjacency matrix for 240 time instants, and we calculated the mean square error between real and simulated data. This error quantifies how much our model is

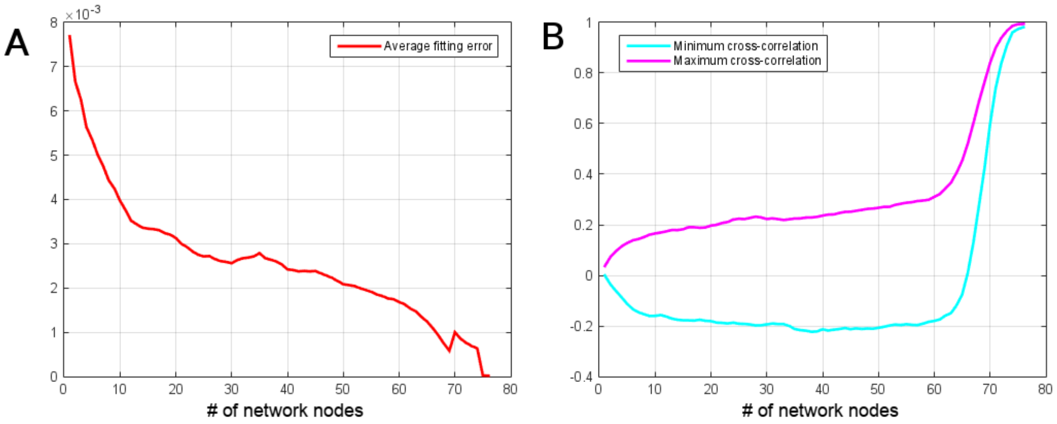

Figure S1: **Performance of the model for different network sizes.** For all subjects involved in the study, the adjacency matrix of the model has been estimated by using an increasing number of nodes (from 2 to 77). In (**A**), the average fitting error between simulated and real data is shown, while in (**B**), the minimum (cyan) and maximum (magenta) cross-correlation curves between real and simulated data are reported.

able to predict the brain dynamics using a reduced number of samples N_E. For each size of the estimation set N_E, we evaluated the mean, standard deviation, best and worst cases obtained within the group of available subjects under investigation. Moreover, we counted the number of outliers N_O within this groups, as the number of subjects whose prediction error is bigger than the mean plus 3 times the standard deviation.

Afterwards, we performed a similar procedure based on a linear system, defined as:

$$\dot{x} = \mathbf{M}x. \tag{8}$$

This time, we estimated the dynamical matrix $\mathbf{M}$ using the same approach employed for the signed EGN-B connectivity matrix $\mathbf{A}'$.

The results obtained using the two models are reported in Table S1. Dark blue lines refer to the results obtained with the EGN-B model, while light blue lines reports the results for the linear model. The mean error is always lower when the EGN-B is used to predict the brain dynamics of the subjects (except for the case with $N_E = 150$). The mean obtained using the EGN-B model does not present strong differences when N_E varies - its order of magnitude is always 10^{-2} - as it happens for the linear model. Moreover, the results obtained using the proposed model are more robust than the linear case: indeed the latter shows highly variable performances compared to the the EGN-B model (see the standard deviation and worst case columns of Table S1). Finally, the number of outliers in the investigated groups is always null for the EGN-B case when the estimation set is small ($N_E \leq 140$) compared with the linear case when there is always at least one outlier.

N_E	Mean	Std	WC	BC	N_O
70	0.05	0.02	8.90e-2	14.00e-3	0
	1.77	4.46	18.30	5.33e-3	2
80	0.05	0.02	8.03e-2	10.40e-3	0
	3.49	11.27	61.20	6.94e-3	1
90	0.05	0.02	8.72e-2	6.00e-3	0
	2.18	10.39	65.60	6.51e-3	1
100	0.04	0.02	7.60e-2	7.02e-3	0
	2.26	13.47	85.30	5.23e-3	1
110	0.04	0.02	7.32e-2	6.17e-3	0
	0.16	0.58	3.67	4.98e-3	1
120	0.04	0.02	7.68e-2	5.24e-3	0
	0.82	4.94	31.30	4.14e-3	1
130	0.03	0.02	5.40e-2	5.34e-3	0
	0.05	0.20	1.26	3.85e-3	1
140	0.03	0.02	8.43e-2	4.49e-3	0
	0.94	5.57	35.30	4.24e-3	1
150	0.03	0.03	12.20e-2	3.61e-3	1
	0.01	0.02	0.11	3.52e-3	1
160	0.03	0.02	11.20e-2	3.33e-3	1
	16.01	100.86	638.00	2.96e-3	1
170	0.02	0.03	11.40e-2	2.44e-3	1
	1.51	9.43	59.70	2.25e-3	1

Table S1: **Comparison between EGN-B and linear model based on prediction error.** For each size of the estimation set N_E, we report the mean, standard deviation (Std), the worst case (WC), the best case (BC) and the number of outliers N_O within the group of available subjects under investigation. The results obtained using the EGN-B and the linear model are reported in the dark blue lines and light blue lines, respectively.

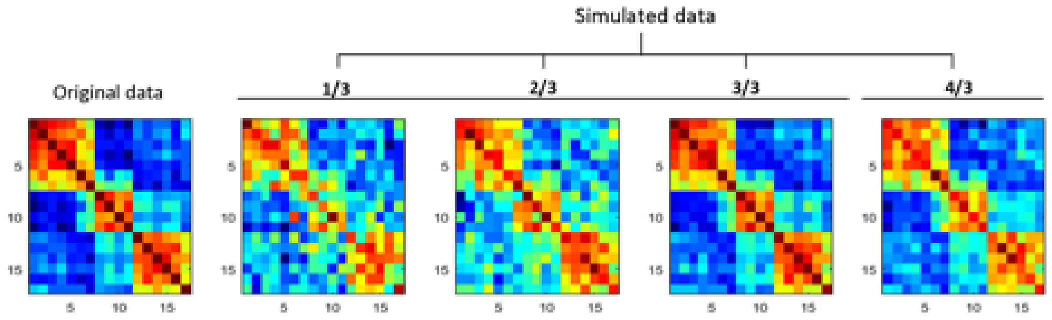

Figure S2: **Functional connectivity (correlation) simulation and temporal prediction.** Functional connectivity matrices built using an incremental amount of available data points are shown (1/3, 2/3, 3/3), leading to a matrix almost identical to the original one when 100% of the data are modeled (3/3). Interestingly, the application of EGN-B on the entire set of time points also allows to simulate data beyond the available dataset, as in the case of the connectivity matrix on the far right (4/3), showing changes in future network behavior by simulating an additional 33% of data points.

References

[1] Bonavita, S. *et al.* Default mode network changes in multiple sclerosis: a link between depression and cognitive impairment? *Eur. J. Neurol.* **24,** 27-36 (2017).

[2] Zhang, Y., Brady, M., Smith, S. Segmentation of brain MR images through a hidden Markov random field model and the expectation-maximization algorithm. *IEEE T. Med. Imaging* **20,** 45-57 (2001).

[3] Bassett, D. *et al.* Hierarchical organization of human cortical networks in health and schizophrenia. *J. Neurosci.* **28,** 9239-9248 (2008).

[4] Madeo, D., Mocenni, C. Game interactions and dynamics on networked populations. *IEEE T. Automat. Contr.* **60,** 1801-1810 (2015).

[5] Ljung, L. *System identification - Theory for the User* (Prentice-Hall, 1999).
